# Comment on “Cerebral pulse oximetry in carotid endarterectomy: an impact study”

**DOI:** 10.1590/1806-9282.20251716

**Published:** 2026-05-11

**Authors:** Hüseyin Durmaz

**Affiliations:** 1 Konya City Hospital – Konya, Turkey.

Dear Editor,

I read with interest the article “Cerebral pulse oximetry in carotid endarterectomy: an impact study” recently published in the journal Revista da Associação Médica Brasileira by Dr. Yürük et al^1^. This study is an important and timely study evaluating the early clinical outcomes achieved with cerebral pulse oximetry and its role in the assessment of procedure-related episodes.

The data presented in the study clearly demonstrate the impact of cerebral pulse oximetry on patient morbidity. The recommendations offered to improve the predictability of these systematic clinical outcomes and procedure-related components are quite important. However, several points need to be addressed.

Cerebral pulse oximetry reflects the distribution of chromophores composed of hemoglobin and cytochrome c oxidase, thus allowing for intraoperative insight into cerebral saturation^2^. Patient hemoglobin data were not available in the study. Hemoglobin deterioration has the potential to alter cerebral pulse oximetry, as cerebral pulse oximetry can be altered.

Perioperative blood exchanges can alter cerebral pulse oximetry values^3^. The study did not address the relationship between perioperative systemic blood pressure and cerebral pulse oximetry, which could affect the results.

In conclusion, Dr. Yürük et al.’s study provides a valuable starting point by addressing the important contributions of cerebral pulse oximetry to surgery. They eagerly await further research in this area and state that the authors will address the lack of consideration of these recommendations in their study.

## Data Availability

The datasets generated and/or analyzed during the current study are available from the corresponding author upon reasonable request.
